# Mucosal-Associated Invariant T Cells Display Diminished Effector Capacity in Oesophageal Adenocarcinoma

**DOI:** 10.3389/fimmu.2019.01580

**Published:** 2019-07-10

**Authors:** Ashanty M. Melo, Aisling M. O'Brien, James J. Phelan, Susan A. Kennedy, Nicole A. W. Wood, Natacha Veerapen, Gurdyal S. Besra, Niamh E. Clarke, Emma K. Foley, Akshaya Ravi, Finbar MacCarthy, Dermot O'Toole, Narayamasami Ravi, John V. Reynolds, Melissa J. Conroy, Andrew E. Hogan, Jacintha O'Sullivan, Margaret R. Dunne

**Affiliations:** ^1^Department of Surgery, Trinity Translational Medicine Institute, St. James's Hospital, Dublin, Ireland; ^2^Childhood Obesity Research Group, National Children's Research Centre, Dublin, Ireland; ^3^School of Biosciences, University of Birmingham, Birmingham, United Kingdom; ^4^Department of Clinical Medicine, Trinity Translational Medicine Institute, Trinity College Dublin, Dublin, Ireland; ^5^National Oesophageal and Gastric Centre, St. James's Hospital, Dublin, Ireland; ^6^Obesity Immunology Research Group, Human Health Institute, Maynooth University, Co Kildare, Ireland

**Keywords:** MAIT cells, oesophageal adenocarcinoma, Barrett's Oesophagus, cancer immunology, tumor microenvironment, PD-1

## Abstract

Oesophageal adenocarcinoma (OAC) is an aggressive malignancy with poor prognosis, and incidence is increasing rapidly in the Western world. Mucosal-associated invariant T (MAIT) cells recognize bacterial metabolites and kill infected cells, yet their role in OAC is unknown. We aimed to elucidate the role of MAIT cells during cancer development by characterizing the frequency, phenotype, and function of MAIT cells in human blood and tissues, from OAC and its pre-malignant inflammatory condition Barrett's oesophagus (BO). Blood and tissues were phenotyped by flow cytometry and conditioned media from explanted tissue was used to model the effects of the tumor microenvironment on MAIT cell function. Associations were assessed between MAIT cell frequency, circulating inflammatory markers, and clinical parameters to elucidate the role of MAIT cells in inflammation driven cancer. MAIT cells were decreased in BO and OAC blood compared to healthy controls, but were increased in oesophageal tissues, compared to BO-adjacent tissue, and remained detectable after neo-adjuvant treatment. MAIT cells in tumors expressed CD8, PD-1, and NKG2A but lower NKG2D than BO cohorts. MAIT cells produced less IFN-γ and TNF-α in the presence of tumor-conditioned media. OAC cell line viability was reduced upon exposure to expanded MAIT cells. Serum levels of chemokine IP-10 were inversely correlated with MAIT cell frequency in both tumors and blood. MAIT cells were higher in the tumors of node-negative patients, but were not significantly associated with other clinical parameters. This study demonstrates that OAC tumors are infiltrated by MAIT cells, a type of CD8 T cell featuring immune checkpoint expression and cytotoxic potential. These findings may have implications for immunotherapy and immune scoring approaches.

## Introduction

Mucosal-associated invariant T (MAIT) cells are a population of unconventional or innate-like T cells, characterized in humans by expression of a semi-invariant T cell receptor Vα7.2-Jα33 chain, and high expression of the C-type lectin CD161 (NKR-P1A) (1, 2). Human MAIT cells comprise 1–5% of T cells in healthy blood, are abundant in the mucosa and particularly in the liver, where they can comprise up to half of all T cells (3). MAIT cells recognize microbe-derived vitamin B metabolites presented by the MHC-Ib-related protein MR1 (4, 5). MAIT cells can detect and lyse microbially-infected cells and are therefore, thought to play a putative immunosurveillance role in the mucosa (6). Upon *in vitro* activation, MAIT cells produce lytic granules such as granzymes and cytokines such as IFN-γ, TNF-α, and IL-17 (3). MAIT cells have been implicated in the pathology of several inflammatory diseases such as inflammatory bowel diseases (7), rheumatoid arthritis (8), systemic lupus erythematosus (9), type I diabetes (10), and multiple sclerosis (11, 12), yet their role in cancer is less clear.

Mucosal-associated invariant T (MAIT) cells have been detected within many tumor types, including gastric, lung, breast, liver, thyroid, colorectal, kidney, brain, and multiple myeloma (3, 13–18). MAIT cells are reportedly decreased in the circulation of patients with colorectal cancer compared to healthy controls, and are found at elevated levels in tumors, compared to adjacent non-tumor tissue and normal tissue (14, 16, 17). A standing question in the cancer field is whether MAIT cells share the potent anti-tumor capabilities displayed by other unconventional T cells, such as invariant natural killer T (iNKT) cells and gamma delta (γδ) T cells (19). MAIT cells possess the pre-requisite cytolytic machinery for granule exocytosis, expressing, and granzymes, and perforin (20, 21). Activated MAIT cells inhibit the growth of colorectal cancer cell lines (17) and demonstrate cytotoxic activity comparable to that of natural killer cells, in *in vitro* experiments using multiple myeloma target cells (18). Despite this, MAIT cell abundance in colorectal tumors has been associated with poorer survival outcomes (15) and levels of serum carcinoembryonic antigen (CEA), a protein used to measure cancer progression (17). MAIT cell levels in the blood of patients with mucosal cancers are negatively associated with serum CEA level and tumor nodal stage (16). So whether MAIT cells act as cytolytic anti-tumor effector cells within the tumor microenvironment, or whether their function is subverted into a pro-tumor phenotype, remains to be determined.

Characterization of the frequency and phenotype of tumor-infiltrating lymphocytes (TIL) has revealed prognostic roles for certain cells in solid tumors in recent years, particularly CD8^+^ T cells (22–24). Such studies strongly indicate that unconventional T cells in particular may play a more important role in anti-tumor immunity than originally thought (22). One particularly intriguing finding was that KLRB1, the gene encoding the CD161 molecule, is one of the strongest favorable prognostic markers in solid tumors (22). Although expressed by many leukocytes, MAIT cells express particularly high levels of CD161, warranting further investigation of these cells in the cancer setting (25).

This study aimed to assess the frequency and function of MAIT cells in the setting of oesophageal adenocarcinoma (OAC). OAC is an aggressive malignancy with poor prognosis and is one of the fastest growing malignancies in the Western world (26–28). The 5 year survival for OAC is typically <15% and neo-adjuvant treatment approaches using multi-modal chemotherapy or chemoradiotherapy only result in complete pathological response for a minority (20–30%) of patients (29, 30). OAC is an inflammation-driven cancer, linked with gastroesophageal reflux disease (GORD) and is strongly associated with obesity (31–33). GORD drives establishment of Barrett's oesophagus (BO), a metaplastic disorder where squamous cells of the oesophagus are replaced with intestinal-type columnar cells in response to chronic exposure to stomach acid. A progressive accumulation of genetic mutations then allows for progression from non-dysplastic BO to a state of low grade dysplasia, high grade dysplasia, and eventually, invasive OAC (34). BO is a prime risk factor for OAC development, and therefore, represents a useful pre-neoplastic model to evaluate cellular changes in inflammation-driven cancer development (35). We used this model to study MAIT cells across the progression sequence from inflammation to cancer. MAIT cell frequency, phenotype, and functions were assessed in blood and tissues collected from healthy donors, and BO and OAC patients. MAIT cell frequency was also assessed after neo-adjuvant treatment with either chemotherapy or chemoradiotherapy. In this post-treatment cohort, tissue was also available from the omentum and liver as well as blood and tumors, allowing assessment of MAIT cell frequency in different anatomical sites. Cell function was assessed by analyzing intracellular cytokine production and cytotoxic capability of MAIT cells activated in the presence or absence of conditioned media (TCM) generated from OAC tumor explant tissue, to model the effects of the soluble tumor microenvironment. MAIT cell frequency was assessed with respect to serum inflammatory protein levels and patient clinical parameters; such as tumor stage, nodal involvement, treatment response, and overall survival.

## Materials and Methods

### Ethical Approval

Ethical approval was granted from the St. James's Hospital and Adelaide, Meath, and National Children's Hospitals Research Ethics Committee (SJH/AMNCH, reference number 041113/10804). All specimens were collected with prior informed consent, from patients attending St. James's Hospital or from healthy age-matched donors. This study was carried out in accordance with the World Medical Association's Declaration of Helsinki guidelines on medical research involving human subjects.

### Specimen Collection

Whole blood was collected in EDTA Vacutainer tubes (BD Biosciences) from healthy control donors (*n* = 14; 8 male; mean age 57.4 years [range 42–64 years]), patients with BO (*n* = 35; 27 male; age 60[31–78]) and patients with OAC (*n* = 79; 70 male; mean age at diagnosis 65.1[28–92]). Oesophagogastric tissue biopsies were obtained from patients undergoing endoscopy at St. James's Hospital, from patients with BO (*n* = 33; 23 male; age 58.9[25–76]) and patients with OAC (*n* = 47; 42 male, age 64.2[28–89]). Barrett segment length was measured as per the Prague classification system, with an average length 5.1 cm (range 1–12 cm) and the majority of BO biopsies (27/33) were assigned a Vienna grade of 1 or 2. Demographic information for the pre-treatment OAC cohort is shown in Table 1. All biopsies from this cohort were treatment-naive at the time of collection and diagnoses were subsequently confirmed by histopathological evaluation. Specimens returning an alternative diagnosis (e.g., squamous cell carcinoma (SCC) or gastric cancer) were removed from the final cohort. Post-treatment specimens (blood, omentum, liver, tumor) were also collected from a cohort of *n* = 24 unmatched OAC patients after neo-adjuvant chemotherapy (*n* = 12) or chemoradiotherapy (*n* = 12) treatment at the time of surgery.

**Table 1:** OAC cohort demographics.

	**Biopsies (*n* = 47)**	**Blood (*n* = 79)**
Gender (M:F)	42:5	70:9
Median age (range), years	64.2 (28–89)	65.1 (28–92)
**HISTORY OF BARRETT'S OESOPHAGUS**		
Yes	14	29
No	17	20
Unknown/Not reported	16	30
**TUMOR LOCATION**		
Type I	21	43
Type II	9	11
Type III	11	16
Not reported	6	9
**TUMOR STAGE (CLINICAL)**		
Tx	0	2
T0	0	1
T1	2	8
T2	4	12
T3	34	45
T4	1	1
Not reported	6	10
**NODAL STAGE (CLINICAL)**		
NX	1	2
N0	16	33
N1	15	19
N2	4	6
N3	5	8
Not reported	6	11
**METASTATIC STAGE (CLINICAL)**		
Mx	5	30
M0	12	10
M1	4	7
Not reported	26	31
**PATHOLOGICAL DIFFERENTIATION GRADE**		
Well	2	3
Moderate	15	37
Poor	30	36
Not reported	0	3
**POST-BIOPSY TREATMENT**		
Neo-adjuvant CROSS	13	23
Neoadjuvant MAGIC	12	27
Other treatment	15	11
Surgery only	3	4
Palliative care	3	6
Unknown	1	8
**MANDARD TUMOR REGRESSION GRADE**		
TRG 1	2	1
TRG 2	2	10
TRG 3	5	10
TRG 4	8	15
TRG 5	7	8

### Whole Blood Staining

Fluorochrome-conjugated antibodies were added to 100 μl blood at pre-optimized concentrations and incubated for 15 min at room temperature in the dark. Red cells were lysed using BD Lysing Solution (BD Biosciences, UK), according to manufacturer's recommendations and cells were washed twice in PBS containing 2% fetal calf serum and 0.02% v/v sodium azide (PBA solution). Cells were fixed for 15 min in 1% paraformaldehyde solution (PFA) (Santa Cruz Biotechnology, USA) prior to flow cytometric analysis.

### Tissue Dissociation

Oesophagogastric tissue biopsies of approximately 2-3 mm^3^ in size were collected in saline-soaked sterile gauze for transport to the laboratory, immediately transferred to a complete RPMI 1640 solution (Gibco, UK) with Glutamax, supplemented with 10% v/v fetal calf serum, 100 U/ml Penicillin/Streptomycin, 130 U/ml collagenase type IV (all from Sigma-Aldrich). Tissue biopsies were incubated in this collagenase solution at 37°C for no longer than 30 min, on a shaking incubator set to maximum agitation. The resulting single cell suspension was then washed through a 40 μM sterile filter (BD Biosciences, UK) and cells were washed twice in PBA solution. Cells were resuspended in 100 μl PBA, fluorochrome-conjugated antibodies were added and incubated for 15 min in the dark at room temperature. Cells were washed in PBA solution and fixed for 15 min in 1% PFA solution prior to analysis by flow cytometry. Post-treatment liver and omental specimens were cut into small pieces using a sterile scalpel on a Petri dish, followed by enzymatic digestion for 20 min at 37°C in complete RPMI containing collagenase IV and II, respectively (Sigma-Aldrich, USA). Cells were then flushed through a 40 μM sterile filter, centrifuged at 1,500 rpm for 10 min and any floating adipocytes were removed from the tube with the supernatant, the remaining pellet was washed twice with complete RPMI, prior to staining for flow cytometry.

### Flow Cytometry Phenotyping

The following fluorochrome-conjugated antibodies were used to identify and characterize MAIT cell populations by multi-color flow cytometry: Vα7.2 FITC (clone REA179, Miltenyi Biotec), NKG2A PE (clone REA110, Miltenyi Biotec), CD3 PerCP (clone BW264/56, Miltenyi Biotec), NKG2D PeVio770 (clone BAT221, Miltenyi Biotec), CD161 Brilliant Violet (BV)-421 (clone HP-3G10, BioLegend), CD8 BV510 (clone SK1, BioLegend), PD-1/CD279 APC-Cy7 (clone EH12.2H7, BioLegend). MAIT cells were defined as CD3^+^/Vα7.2^+^/CD161^high^ lymphogated cells. Cells were acquired on a CyAn ADP cytometer (Beckman Coulter) using Summit software (Version 4.0). Gate limits were determined by using fluorescence minus one (FMO) controls. Data was analyzed using FlowJo software, Version 10 (FlowJo, LLC).

### Tumor Conditioned Media Preparation

Oesophageal adenocarcinoma tumor explants of ~2–3 mm^3^ were transferred into 1 ml of complete M199 (cM199) medium (Gibco), made up of M199 supplemented with 1 μg/ml insulin (Sigma) and 10% FCS (Gibco), at one biopsy per well of a 12 well-plate for 24 h at 37°C, 5% CO_2_. The resulting tumor conditioned media (TCM), or media only (no tumor, cM199 only control) wells were harvested, snap frozen in liquid nitrogen and stored at −80°C until required for experimentation.

### Intracellular Cytokine Analysis

Peripheral blood mononuclear cells (PBMC) were prepared from whole blood collected in EDTA Vacutainer tubes (BD Biosciences) by density gradient centrifugation over Lymphoprep^TM^ (Stemcell Technologies) from *n* = 4 healthy donors. PBMC were plated at a concentration of 2 × 10^6^ cells per ml in cRPMI media and stimulated using a T cell activation/expansion kit (Miltenyi Biotec) composed of bead particles bound with biotinylated antibodies specific for CD2, CD3, and CD28 (at concentration recommended by manufacturer), and with recombinant human cytokines IL-12 (50 ng/ml, R&D Systems) and IL-18 (50 ng/ml, R&D Systems). PBMC were stimulated in the presence of 50% TCM from stage III OAC patients, or with 50% cM199 medium only control. Each PBMC donor received TCM from at least two different donors, with a total of six TCM donors was used in total. Media controls were derived from the same cM199 batch as TCM and received the same handling as TCM, with the exception of tumor exposure. PBMC were treated with brefeldin A (eBiosciences) (at 1× concentration, as per manufacturer's recommendation) and cells were incubated overnight (18 h) at 37°C, 5% CO_2_. Cells were harvested, washed in PBA solution and accumulated intracellular cytokines were measured using a Fixation/Permeabilisation Solution kit (BD Biosciences), as per manufacturer's recommendations. In brief, cells were stained with cell surface antibodies (PD-1 PE, CD8 PerCP, Vα7.2 PEVio770, CD3 VioGreen, CD161 APC), (Miltenyi Biotec), were washed, permeabilised, and then stained for intracellular cytokines (IFN-γ FITC, IL-17A VioBlue, and TNF-α APCCy7), (Miltenyi Biotec). Cells were acquired on a FACS Canto flow cytometer (BD Biosciences). Gating cut-offs for cytokine production were determined using unstimulated controls.

### Generation of Expanded MAIT Cells

Peripheral blood mononuclear cells from healthy donors were cultured with vitamin B metabolite antigen 5-ARU (1 μg/ml) and the intermediate methylglyoxal (100 μM) and maintained in complete RPMI media supplemented with IL-2 (100 U/ml). MAIT cells were expanded for an average of 10 days prior to harvesting. MAIT cells were removed from IL-2 media for 24 h prior to experimentation. The purity of expanded MAIT cells ranged from 73 to 92%.

### Cytotoxicity Assay

The oesophageal adenocarcinoma cell line OE33, obtained from the European Collection of Authenticated Cell Cultures (ECACC), was used as a cell target, and expanded MAIT cells were used as effector cells. Expanded MAIT cells were stimulated prior to co-incubation, using TCR beads (T cell activation/expansion kit, Miltenyi Biotec) with and without added cytokines IL-12 (50 ng/ml, R&D Systems) and IL-18 (50 ng/ml, R&D Systems), or were given media only mock stimulation. MAIT cells were then washed prior to co-incubation to remove stimulators. OE33 cells were seeded at a density of 2 × 10^6^ cells/ml in 24 well-plates. Cells were co-incubated at an effector:target ratio of 10:1 for 4 h at 37°C, 5% CO_2_, with or without TCM from a stage 3 OAC donor. Incubation of OE33 cells with 4 μg/mL camptothecin for 4 h was used to create an apoptosis-positive control. For the generation of necrosis positive controls, target cells were incubated at 56°C for 6 min. OE33 target cells were then harvested from wells by trypsinisation. Target cells were identified using CFSE labeling, and cell death was measured by expression of 7-AAD and annexin V, using the Total Cytotoxicity and Apoptosis Detection Kit (BioLegend). Cells were acquired on a FACSCanto II flow cytometer (BD Biosciences).

### Multiplex ELISA Analysis of Serum Proteins

Levels of 54 serum proteins were quantified using a V-plex Human Biomarker 54-plex kit (Meso Scale Diagnostics), as per manufacturer's recommendations. Proteins analyzed included; CRP, Eotaxin, Eotaxin-3, FGF, Flt-1/VEGFR-1, GM-CSF, ICAM-1, IFN-γ, IL-10, IL-12/IL-23p40, IL-12p70, IL-13, IL-15, IL-16, IL-17A, IL-17A/F, IL-17B, IL-17C, IL-17D, IL-1RA, IL-1α, IL-1β, IL-2, IL-21, IL-22, IL-23, IL-27, IL-3, IL-31, IL-4, IL-5, IL-6, IL-7, IL-8, IL-8 (HA), IL-9, IP-10, MCP-1, MCP-4, MDC, MIP-1α, MIP-1β, MIP-3α, PlGF, SAA, TARC, Tie-2, TNF-α, TNF-β, TSLP, VCAM-1, VEGF-A, VEGF-C, VEGF-D. Levels of circulating proteins were compared to percentage MAIT cell data available for *n* = 26 OAC patient blood samples, *n* = 16 OAC tumor samples, and a subset of *n* = 12 OAC patients for whom both matched blood and tumor MAIT cell percentages were available, and Spearman (r) correlation values were calculated using GraphPad Prism (Version 5). Correlation values of >0.6/<-0.6 were considered strongly correlated. Analytes below the kit detection range were not included.

### Statistical Analyses

All statistical tests were carried out using Prism GraphPad, version 5.01. Normality testing (using Kolmogorov-Smirnov, D'Agostino and Pearson, and Shapiro-Wilk tests) showed that most populations were not normally distributed, therefore, non-parametric tests were used, as detailed in figure legends. For multivariate analysis, data were analyzed using SPSS version 24 (IBM, New York, USA). Kaplan Meier estimates were used to calculate survival curves, differences in survival curves were calculated using log-rank analysis. Cox regression multivariate analysis was used to determine independent predictors of survival; only variables with significance on univariate analysis were input into the multivariate analysis. Statistical significance was defined by *p* < 0.05.

## Results

### MAIT Cells Are Reduced in Circulation and Increased in Oesophageal Tissues in Inflammation and Cancer

**Figure 1:**
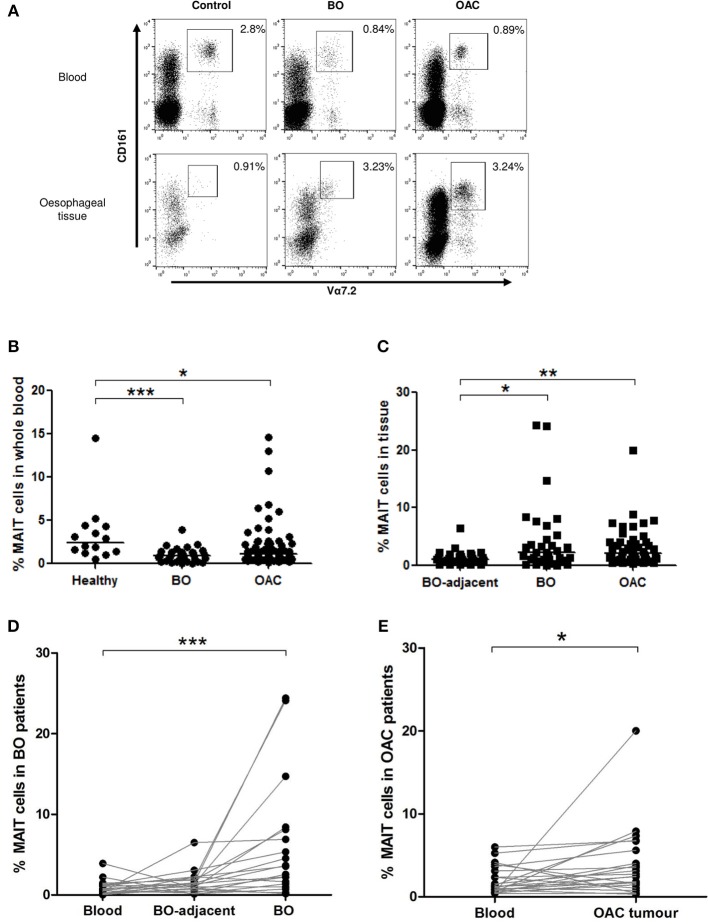
MAIT cell frequency is reduced in blood but enriched in tissues. MAIT cell frequencies were analyzed by flow cytometry in whole blood and collagenase-digested oesophageal tissues, defined as CD161^high^/Va7.2^+^ cells, expressed as a percentage of CD3^+^ lymphocyte gate, as shown in dot plots from representative donor tissue compartments **(A)**. **(B)** MAIT cells were more abundant in whole blood taken from healthy (*n* = 14) age-matched donors, than patients with BO (*n* = 33, *p* < 0.001) or OAC (*n* = 79, *p* < 0.05). **(C)** When compared to BO-adjacent Oesophageal tissue (*n* = 31), MAIT cell frequency was higher in OAC tumor biopsies (*n* = 47, *p* < 0.01) and BO lesions (*n* = 32, *p* < 0.05), respectively. **(D)** MAIT cells in a cohort of matched BO patients were elevated in BO lesions compared to blood (*p* < 0.001, *n* = 20). **(E)** MAIT cells in a cohort of matched patients were higher in OAC tumors compared to blood (*p* < 0.05, *n* = 23). Data was analyzed using a Kruskal-Wallis test followed by Dunn's Multiple Comparison Test. For matched data sets, a Friedman test was used to analyse three matched datasets, and a Wilcoxon signed rank test was used to compare two datasets, respectively, followed by Dunn's Multiple Comparison Test. Horizontal bars indicate median values. *p < 0.05, **p < 0.01, ***p < 0.001.

Mucosal-associated invariant T cells were defined as the CD161^high^/Vα7.2^+^ proportion of the CD3^+^ lymphogate, as shown in representative flow cytometry dot plots (Figure 1A). Doublet exclusion was also performed as part of the gating strategy. MAIT cells were decreased in the circulation of patients with BO (mean 0.86% [range 0.02–2.4%], *n* = 34, *p* < 0.001) and OAC patients (1.9% [0.15–14.6%], *n* = 79, *p* < 0.05) when compared to healthy controls (3.4% [0.48–14.5%], *n* = 14) (Figure 1B). In collagenase-digested Oesophageal tissue suspensions, the percentage MAIT cell frequency was higher in BO lesions (4.38% [0–25%], *n* = 33, *p* < 0.05) and OAC tumors (3.2% [0.32–20%], *n* = 47, *p* < 0.05) when compared to BO-adjacent oesophageal tissues, taken at least 10 cm from the BO lesion (1.34% [0.1–6.49%], *n* = 31) (Figure 1C). These findings were recapitulated in a cohort of matched patient tissues, where MAIT cells also represented a higher proportion of T cells in BO lesions (*p* < 0.001, *n* = 20) (Figure 1D), and OAC patients (*p* < 0.05, *n* = 23) (Figure 1E).

### MAIT Cell Frequency in Post-treatment Tissues

**Figure 2:**
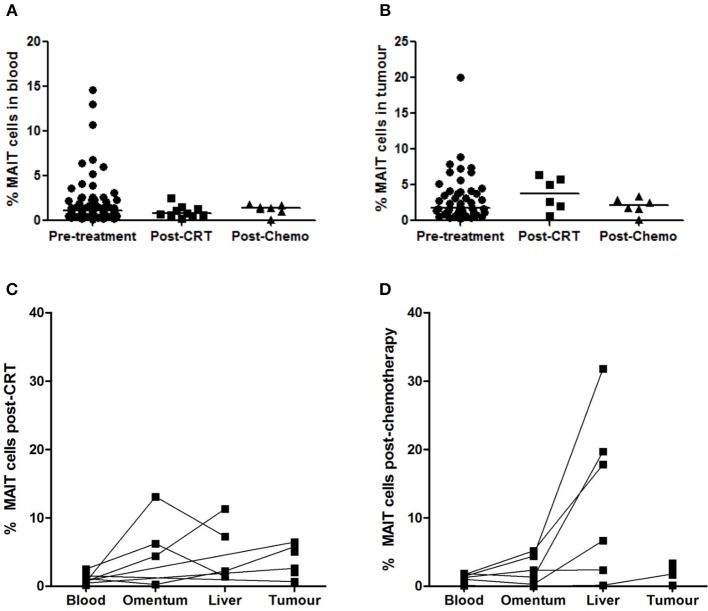
MAIT cell frequencies after neo-adjuvant treatment. MAIT cell frequency was not altered in a cohort of OAC donors after treatment with chemoradiotherapy (CRT) or chemotherapy (chemo), compared to an unmatched pre-treatment patient cohort, in blood **(A)** and tumors **(B)**, as assessed by ANOVA (Kruskal-Wallis test followed by Dunn's Multiple Comparison Test). MAIT cells were well-represented in blood, omentum, liver, and tumors after neo-adjuvant CRT **(C)** and chemotherapy **(D)**. Horizontal bars indicate median values.

Mucosal-associated invariant T cell frequencies in blood and tumors were compared in unmatched cohorts of treatment-naive and neo-adjuvant treated OAC patients. No differences in circulating MAIT cell frequencies were detected when treatment-naive (*n* = 79) and post-chemoradiotherapy (CRT) (*n* = 10) or post-chemotherapy (chemo) (*n* = 7) groups were compared (Figure 2A). Similarly, no difference was detected in tumor MAIT cell levels when pre-treatment (*n* = 47), post-CRT (*n* = 6), and post-chemo (*n* = 6) cohorts were compared (Figure 2B). MAIT cells also remained detectable in the blood (*n* = 10), omentum (*n* = 4), liver (*n* = 4), and tumors (*n* = 6) after treatment with CRT (Figure 2C) or chemo (*n* = 7, 6, 6, 6, respectively), in a partially matched cohort (Figure 2D).

### MAIT Cell Phenotype in OAC Blood and Oesophageal Tissues

**Figure 3:**
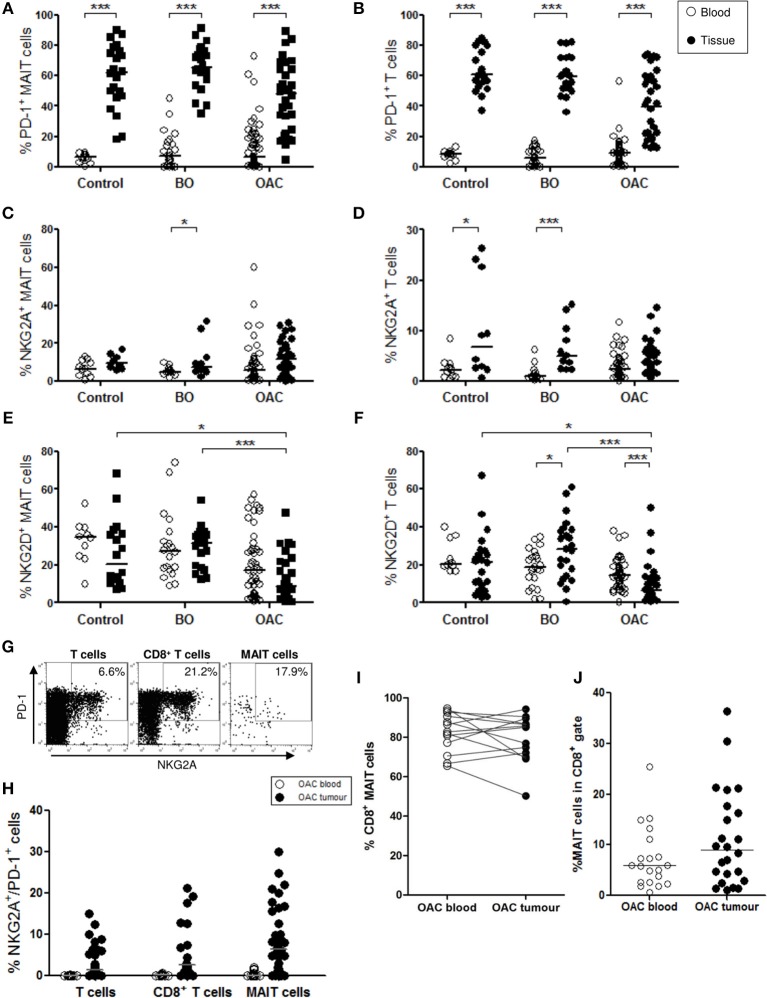
Phenotypic characterization of MAIT cells in blood and oesophageal tissues. MAIT cell (CD3^+^/Vα7.2^+^/CD161^high^) and T cell (CD3^+^) populations were gated and percentage positive expression for PD-1, NKG2A, NKG2D, and CD8 was analyzed in blood (open circles) and oesophageal tissue specimens (filled circles). BO-adjacent oesophageal tissue from *n* = 22 donors was used as a tissue control, and *n* = 11 healthy age-matched donors were used for blood controls. PD-1 expression was higher in all tissue compartments compared to blood, for both MAIT cells **(A)** and T cells **(B)**. Expression of inhibitory marker NKG2A by MAIT cells **(C)** and T cells **(D)**, and costimulatory marker NKG2D **(E,F)**, respectively, for *n* = 23 BO and *n* = 51 OAC blood samples, and *n* = 22 BO and *n* = 32 OAC oesophageal tissue biopsies. **(G)** Representative flow cytometry plots depicting PD-1 and NKG2A co-expression gating for a single representative OAC biopsy donor. **(H)** Co-expression of NKG2A and PD-1 by different OAC T cell compartments. **(I)** CD8 expression by MAIT cells was similarly high in blood and OAC tumor tissue, for *n* = 13 matched OAC donors, and MAIT cells account for a substantial proportion of CD8^+^ T cells in blood and OAC tumors **(J)**. Datasets were analyzed by ANOVA (Kruskal-Wallis test followed by Dunn's Multiple Comparison Test). Horizontal bars indicate median values. *p < 0.05, ***p < 0.001.

Mucosal-associated invariant T cell expression of immune inhibitory markers programmed cell death protein 1 (PD-1) and NKG2A, and costimulatory marker NKG2D, was assessed in the blood of 11 healthy donors, 23 patients with BO, and 58 patients with OAC, and in the oesophageal tissue of 22 BO and 39 OAC patients (Figure 3). BO-adjacent tissue (*n* = 22) was used as control tissue. PD-1 expression was higher (*p* < 0.001) on MAIT cells (Figure 3A) and T cells (Figure 3B) from all oesophageal tissue types (BO-adjacent control, BO, and OAC) when compared to blood, but no differences were detected between tissue types, with uniform high expression observed in all oesophageal tissues. This contrasted with MAIT cell expression of inhibitory receptor NKG2A (Figure 3C) and costimulatory receptor NKG2D (Figure 3E), where expression was largely similar between the blood and oesophageal tissue compartments. Significant blood and tissue expression differences were observed in the T cell compartment however (Figure 3D), with NKG2A expression being higher in BO-adjacent (*p* < 0.05) and BO (*p* < 0.001) tissues compared with whole blood controls. NKG2D expression by MAIT cells was lower in OAC tumor tissue however, compared to BO (*p* < 0.001) and control tissues (*p* < 0.05) (Figure 3E), and this lack of NKG2D in OAC tumors was also observed for T cells (Figure 3F). NKG2D expression on T cells was higher in BO tissue (*p* < 0.05) compared to blood, however the opposite trend was observed in OAC patients, with NKG2D levels being expressed at lower levels in tumor tissue compared to OAC blood (*p* < 0.001).

We also investigated co-expression of inhibitory markers NKG2A and PD-1 in OAC patient blood (*n* = 17) and biopsies (*n* = 35) (Figures 3G,H), and observed that co-expression was higher in tissues, and that MAIT cells co-expressed NKG2A and PD-1 at levels similar to T cells and CD8 T cells (Figure 3H). CD8 expression was also measured on MAIT cell in blood and tissues, and was observed at similar high levels; a mean percentage of 82% in blood and 79% in OAC tissues, for *n* = 13 matched OAC donors (Figure 3I). MAIT cells accounted for a mean percentage of 7% of all circulating CD8^+^ T cells (*n* = 20), and 11% of CD8^+^ TILs (*n* = 24) (Figure 3J).

### The OAC Tumor Microenvironment Reduces MAIT Cell Expression of T_H_1 Cytokines

**Figure 4:**
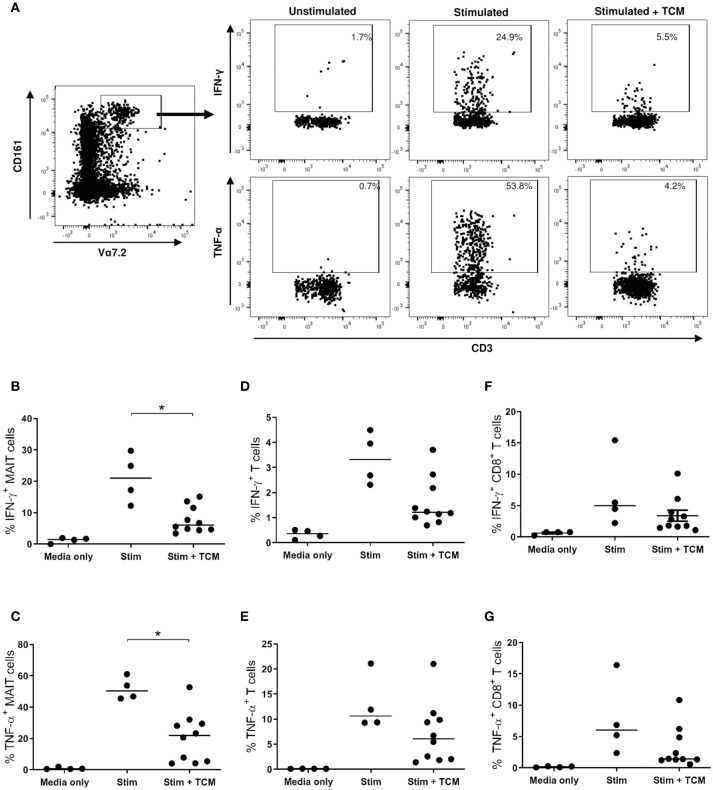
IFN-γ and TNF-α expression is reduced when healthy MAIT cells are activated in the presence of OAC tumor conditioned media. PBMC from *n* = 4 healthy donors received either medium only or TCR and cytokine stimulation in the absence and presence of tumor conditioned medium (TCM) from *n* = 6 stage III OAC patients, and intracellular cytokine production was measured by flow cytometry, as shown for a single representative donor **(A)**. Percentage IFN-γ and TNF-α expression is shown within the MAIT **(B,C)**, T cell **(D,E)**, and CD8^+^ T cell **(F,G)** compartments, respectively. Datasets were analyzed by ANOVA (Kruskal-Wallis test followed by Dunn's Multiple Comparison Test). Horizontal bars indicate median values. *p < 0.05.

Due to the limited size of the oesophageal tissue biopsies available, the effects of the OAC tumor microenvironment on MAIT cell function were evaluated using a tissue explant model. PBMC from *n* = 4 healthy donors were given overnight stimulation with TCR beads and cytokines IL-12 and IL-18 in the presence or absence of TCM from treatment-naïve stage III OAC tumors, and intracellular cytokine production was then assessed by multi-color flow cytometry. Unstimulated PBMC showed no appreciable cytokine production, whereas stimulated cells expressed elevated levels of IFN-γ, TNF-α, and IL-17A, in all cell compartments examined; namely MAIT cells, T cells, and CD8^+^ T cells (Figure 4). PBMC stimulated in the presence of control media (i.e., same media incubated and preserved in the same manner as TCM, but without tumor exposure) showed similar cytokine production profiles as stimulated cRPMI media controls (data not shown). In the presence of OAC TCM, the percentage of MAIT cells expressing IFN-γ (Figure 4B) and TNF-α (Figure 4C) was reduced (*p* < 0.05 for both). No changes were noted in production of IL-17A however, for any T cell subtypes (data not shown). No differences were observed in T cell (Figures 4D,E) or CD8^+^ T cell (Figures 4F,G) cytokine production in the presence of TCM. PD-1 expression was also measured (data not shown), but no changes were observed upon stimulation or TCM addition, for any of the T cell compartments measured.

### Oesophageal Cancer Cell Line Viability Is Reduced in the Presence of Expanded MAIT Cells

**Figure 5:**
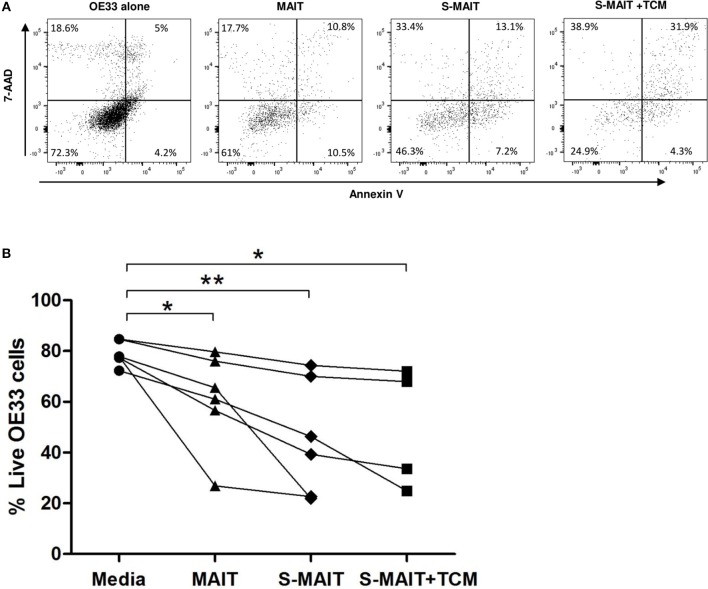
Oesophageal adenocarcinoma cell line viability is reduced after incubation with MAIT cells. Oesophageal cancer cell lines (OE33) were cultured with expanded MAIT cells from *n* = 6 healthy donors, in the presence and absence of MAIT cell stimulation and OAC tumor conditioned media. Cell viability was measured after 4 h by flow cytometric analysis of 7-AAD and Annexin V, with viable cells defined as a double negative population, as shown for a single representative donor **(A)**. Compiled data from *n* = 3 independent experiments showed that OE33 cell viability was reduced in the presence of MAIT cells pre-stimulated with TCR beads and cytokines IL-12 and IL-18 (S-MAIT), however TCM from *n* = 4 OAC patients had no effect on this viability reduction **(B)**. Data analyzed by ANOVA (Kruskal-Wallis test followed by Dunn's Multiple Comparison Test). *p < 0.05, **p < 0.01.

Viability of the OAC tumor cell line, OE33, was reduced after 4 h exposure to expanded MAIT cells, as determined by flow cytometric detection of annexin V and 7-AAD expression (Figure 5A). OE33 cells lacking expression of these markers were defined as viable. MAIT cells were expanded from *n* = 6 healthy PBMC donors, and were co-cultured with OE33 cells, with or without prior stimulation with TCR activation beads and cytokines IL-12 and IL-18. OE33 cell viability was reduced (*p* < 0.05) when co-cultured with unstimulated expanded MAIT cells but was most reduced (*p* < 0.01) when MAIT cells received prior stimulation (Figure 5B). Despite inter-experimental variation observed in OE33 cell viability percentage, this trend of reduced viability in the presence of MAIT cells was consistently seen in all three experimental runs, and for all *n* = 6 expanded MAIT cell donors tested. The addition of TCM derived from *n* = 4 different stage III OAC patients had no effect on MAIT cell cytotoxicity in this experimental system. One expanded MAIT cell donor showed a particularly potent ability to kill the OE33 cells, even in the absence of stimulation. Removal of this dataset does not abrogate the overall significance of this finding.

### MAIT Cells Are Inversely Associated With Circulating Levels of Chemokine IP-10

**Figure 6:**
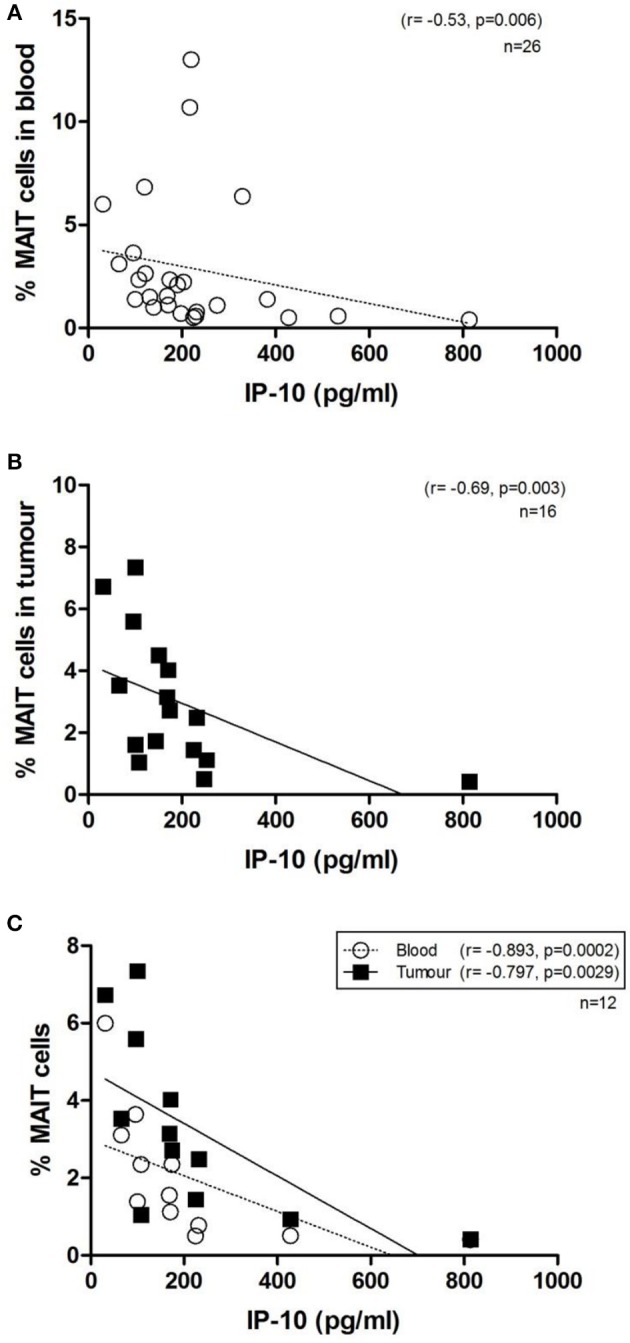
MAIT cell frequency in OAC whole blood and tumors is inversely correlated with circulating levels of IP-10. Serum protein levels were quantified by 54-plex ELISA and correlated with MAIT cell frequencies in OAC patient blood and tumor biopsies. MAIT cell frequency was inversely correlated with levels of circulating IP-10, in *n* = 26 blood donors **(A)**, *n* = 16 OAC tumors **(B)** and *n* = 12 OAC patients with both matched blood and tumor available **(C)**. Correlations were assessed by Spearman *r*-test.

Mucosal-associated invariant T cell frequencies in OAC blood and tumors, expressed as a percentage of T cells, was compared to levels of 54 serum markers of inflammation by multiplex ELISA. Of these, the chemokine interferon gamma-induced protein, IP-10, (also known as CXCL10) was observed to be inversely associated with MAIT cell frequency, in both blood (*n* = 26, *r* = −0.53, *p* = 0.006) and tumors (*n* = 16, *r* = −0.69, *p* = 003) of OAC patients (Figures 6A,B). This negative correlation was also noted in a cohort of *n* = 12 OAC patients with matched blood and tumors, (Figure 6C). Levels of macrophage derived chemokine, (MDC, also known as CCL22) and MIP-1β (CCL4) levels were also observed to be negatively associated with MAIT cell levels, but this correlation was only observed in tumors, but not for blood MAIT cells, as summarized in Table 2.

**Table 2:** Correlations with MAIT cell frequency and serum proteins.

	**Blood (*****n*** **=** **26)**			**Tumor (*****n*** **=** **16)**		
	**Spearman r**	***P*-value**	**95% CI**	**Spearman *r***	***P*-value**	**95% CI**
bFGF	0.05129	0.8035	−0.3536 to 0.4400	0.5118	0.0427	0.005313 to 0.8093
CRP	0.2674	0.1866	−0.1458 to 0.6011	0.07353	0.7867	−0.4511 to 0.5604
Eotaxin	−0.1894	0.354	−0.5460 to 0.2252	−0.3647	0.1649	−0.7362 to 0.1757
Eotaxin-3	0.09061	0.6598	−0.3185 to 0.4713	−0.5118	0.0427	−0.8093 to−0.005313
Flt-1	−0.3892	0.0494	−0.6814 to 0.01002	−0.2294	0.3927	−0.6603 to 0.3151
ICAM-1	0.1077	0.6005	−0.3029 to 0.4846	−0.1441	0.5944	−0.6075 to 0.3924
IFN-γ	−0.2117	0.3098	−0.5685 to 0.2121	−0.4418	0.1138	−0.7943 to 0.1334
IL-10	−0.2456	0.2586	−0.6057 to 0.1979	−0.2724	0.368	−0.7248 to 0.3442
IL-12/IL-23p40	−0.2219	0.2759	−0.5693 to 0.1928	−0.35	0.2009	−0.7389 to 0.2139
IL-15	−0.3444	0.0849	−0.6527 to 0.06172	−0.1059	0.6963	−0.5824 to 0.4248
IL-16	−0.1453	0.4787	−0.5133 to 0.2678	−0.3088	0.2445	−0.7059 to 0.2360
IL-17A	−0.3174	0.1141	−0.6349 to 0.09188	−0.3412	0.1959	−0.7236 to 0.2016
IL-17B	−0.06416	0.7658	−0.4658 to 0.3594	0.04415	0.871	−0.4743 to 0.5399
IL-17D	−0.1705	0.4367	−0.5536 to 0.2721	−0.2393	0.3904	−0.6787 to 0.3263
IL-1RA	−0.3074	0.1266	−0.6283 to 0.1028	−0.09118	0.737	−0.5725 to 0.4369
IL-1α	0.1771	0.419	−0.2658 to 0.5582	0.1319	0.6676	−0.4665 to 0.6475
IL-22	−0.01218	0.955	−0.4241 to 0.4039	−0.411	0.1443	−0.7800 to 0.1701
IL-27	0.04445	0.8293	−0.3596 to 0.4344	0.07647	0.7783	−0.4488 to 0.5625
IL-5	0.1073	0.602	−0.3033 to 0.4843	0.0132	0.9643	−0.5337 to 0.5524
IL-6	−0.1731	0.3978	−0.5340 to 0.2412	−0.2893	0.2957	−0.7066 to 0.2774
IL-7	−0.2212	0.2774	−0.5688 to 0.1934	−0.4059	0.1188	−0.7576 to 0.1284
IL-8	0.1665	0.4162	−0.2475 to 0.5291	0.08214	0.771	−0.4624 to 0.5817
IL-9	0.03509	0.8737	−0.3937 to 0.4514	−0.2044	0.4833	−0.6728 to 0.3810
IP-10	−0.5252	0.0059	−0.7634 to−0.1612	−0.6912	0.003	−0.8875 to−0.2825
MCP-1	−0.2619	0.1962	−0.5974 to 0.1515	−0.1118	0.6803	−0.5863 to 0.4199
MCP-4	−0.3201	0.1109	−0.6367 to 0.08892	−0.4559	0.0759	−0.7825 to 0.06760
MDC	−0.2342	0.2494	−0.5780 to 0.1802	−0.5765	0.0194	−0.8388 to−0.09705
MIP-1α	−0.1068	0.6114	−0.4911 to 0.3123	−0.345	0.208	−0.7363 to 0.2193
MIP-1β	−0.07488	0.7162	−0.4589 to 0.3327	−0.6	0.014	−0.8491 to−0.1326
MIP-3α	−0.05746	0.7804	−0.4449 to 0.3482	0.08824	0.7452	−0.4393 to 0.5705
PlGF	−0.1777	0.3852	−0.5374 to 0.2367	−0.2882	0.279	−0.6944 to 0.2573
SAA	−0.05745	0.7804	−0.4449 to 0.3482	0.07941	0.77	−0.4464 to 0.5645
TARC	−0.1994	0.3289	−0.5532 to 0.2154	−0.4	0.1248	−0.7546 to 0.1353
Tie-2	−0.1746	0.3936	−0.5351 to 0.2397	−0.04706	0.8626	−0.5419 to 0.4721
TNF-α	−0.2575	0.2041	−0.5943 to 0.1561	−0.3	0.2773	−0.7125 to 0.2665
TNF-β	−0.3213	0.1349	−0.6552 to 0.1177	−0.01542	0.9583	−0.5539 to 0.5322
TSLP	−0.06845	0.7743	−0.5066 to 0.3978	−0.4979	0.0833	−0.8290 to 0.09146
VCAM-1	−0.05813	0.7779	−0.4455 to 0.3476	−0.05	0.8541	−0.5440 to 0.4698
VEGF	0.0003419	0.9987	−0.3974 to 0.3979	−0.3824	0.1439	−0.7454 to 0.1557
VEGF-C	−0.04685	0.8202	−0.4364 to 0.3575	−0.08529	0.7535	−0.5685 to 0.4417
VEGF-D	0.08172	0.6915	−0.3265 to 0.4643	−0.09412	0.7288	−0.5745 to 0.4345

### MAIT Cells and Clinical Outcomes

**Figure 7:**
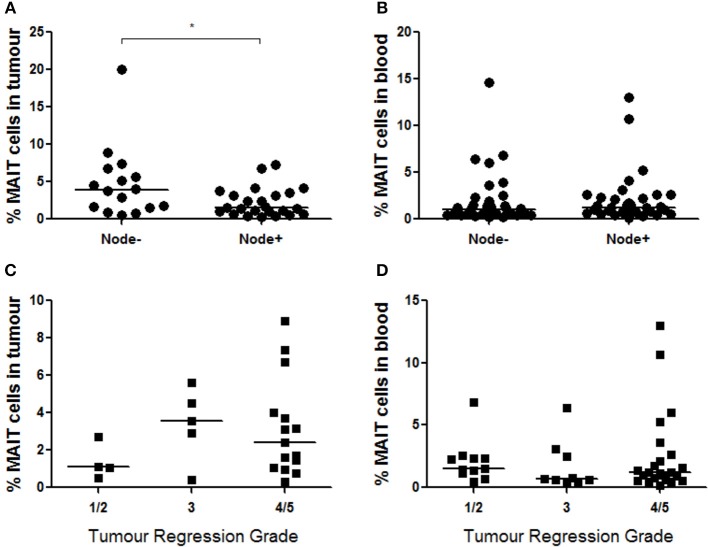
MAIT cells are more abundant in node-negative OAC tumors. **(A)** MAIT cell frequency was assessed in *n* = 40 OAC tumors with respect to nodal status, and node-negative patients displayed higher MAIT cells (*n* = 16, *p* < 0.029) compared to node positive patients (*n* = 24), as assessed by Mann-Whitney *U*-test. **(B)** MAIT cell levels in blood did not show differences when node-negative (*n* = 32) and node-positive (*n* = 33) patients were compared. MAIT cell percentages in were also not different in tumors **(C)** (*n* = 24) or blood **(D)** (*n* = 43) of donors with different TRG scores, as assessed by (Kruskal-Wallis test followed by Dunn's Multiple Comparison Test). Horizontal bars indicate median values. *p < 0.05.

Mucosal-associated invariant T cell frequency in OAC tumors (*n* = 47) was evaluated with respect to various clinical parameters such as clinical and pathological TNM stages, BMI, tumor differentiation state, known history of BO, survival time, and response to neoadjuvant chemoradiotherapy, as reported by Mandard tumor regression grade (TRG). MAIT cells were more abundant in OAC tumors in a cohort of *n* = 16 patients without nodal involvement (*p* < 0.029), compared to *n* = 24 node-positive patients (Figure 7A), although no difference was observed in blood (Figure 7B). Univariate analysis of *n* = 47 OAC tumors showed that increased nodal score is associated with a poorer prognosis (*p* = 0.36, HR = 6.28, 95% CI = 1.13–34.9). Interestingly, the risk of death is further increased in patients where tumor MAIT cell levels are low (p = 0.31, HR = 7.57, 95% CI = 1.21–47.3). No differences were observed in MAIT cell frequency when OAC patients were classified by TRG, with scores of 1–2 indicating a good response to neo-adjuvant treatment, TRG3 indicating no response, and TRG4-5 indicating tumor progression, when tumor MAIT cell frequencies (Figure 7C) (*n* = 24) and blood (Figure 7D) (*n* = 43) were assessed. Survival time was not different between MAIT cell high or low populations, either in tumors (*p* = 0.2384, HR = 1.689, 95% CI = 0.71–4.04) or blood (*p* = 0.2403, HR = 1.433, 95% CI = 0.79–2.61), using median frequency as a cut-off value (data not shown). No other associations were observed.

## Discussion

Over the last decade, the immunological component of the tumor microenvironment has been shown to be of great prognostic importance in many gastrointestinal cancers, allowing prediction of patient survival, recurrenc, and even response to treatment (22, 24, 36–38). The advent of the Immunoscore method for predicting patient clinical outcomes based on immunohistochemical analysis of T cell subsets has, along with other studies, highlighted the importance of CD8 T cells in optimal anti-tumor immune responses (37), and the success of immune checkpoint inhibitor therapies has only reinforced the urgent need for a greater understanding of the immune response to cancer (39), particularly in cancers with poor survival outcomes, such as OAC. We are interested in elucidating the roles played by unconventional, innate-like T cells, many of which also express CD8 and possess CTL function, such as MAIT cells.

This study shows for the first time that MAIT cells comprise a significant proportion of T cells in human Oesophageal mucosa; in inflamed BO lesions, BO-adjacent regions, and in OAC tumor tissues. Furthermore, we observed that MAIT cells account for up to 35% of tumor infiltrating CD8^+^ T cells in OAC tumors. In agreement with other studies in mucosal cancers (16, 17), we observed that MAIT cells were more abundant in tumor tissues than in circulation. MAIT cells accounted for a similar proportion of total T cells at BO and tumor sites, despite the observation that T cells comprised a more abundant proportion of the lymphogate in tumors compared to BO tissue (data not shown), meaning that MAIT cells were equally represented in both inflammatory and cancer states. In addition to the OAC cohort, we observed that MAIT cells were also decreased in the blood of patients with SCC of the oesophagus (*p* < 0.01, *n* = 11), but not in a cohort of patients with gastric cancer (*n* = 7), when compared to healthy controls (data not shown). A decrease in circulating MAIT cells is commonly reported in cancer and other diseases (17, 40–42), hypothesized to reflect homing to active sites of disease, and therefore, potential MAIT cell involvement. However, MAIT cells normally comprise a significant proportion of T cells in the liver and mucosal tissues, and many factors can potentially influence their abundance. We observed that MAIT cells remain detectable in blood, omentum, liver, and tumor tissues after neo-adjuvant treatment with chemotherapy or chemoradiotherapy regimens, and that MAIT cell frequency in whole blood and tumor tissue was not altered after chemoradiotherapy, in agreement with other reports that MAIT cells are unaffected by chemotherapy in breast cancer (3). Differences in the oesophageal microbiome have been reported in patients with GORD and BO, who show a broader range of bacterial species predominated by Gram-negative bacteria, whereas the normal microbiome is predominated by Gram-positive bacteria (43). Oesophageal colonization by *Campylobacter concisus* has been reported in OAC, accompanied by an increase in the MAIT cell activating cytokine, IL-18 (44). Therefore, it is possible that MAIT cell abundance in the Oesophageal mucosa could be influenced by microbial as well as immunological or disease-related factors.

T cells have previously been implicated in the establishment of oesophageal inflammation, since their infiltration into oesophageal tissue precedes tissue damage (45) and inflammation (46). We therefore, examined the phenotypic and functional features of MAIT cells in healthy, inflamed non-cancerous, and cancerous states. We observed that PD-1 expression was high on all tissue T cell subsets examined, in all tissue types, a feature seemingly common in tumor associated T cells (14, 47–49). Elevated PD-1 expression is associated with an exhausted phenotype in CD8^+^ TIL, characterized by poor proliferation, cytokine production, and effector function (48). However, some studies indicate that unconventional T cells can retain cytotoxic ability despite PD-1 expression (50). We did not detect any appreciable expression of exhaustion marker LAG-3 (data not shown). We did observe that expression of the natural cytotoxicity receptor NKG2D was lowest in OAC tissue (Figure 3E), further indicating a potential loss of effector function, as also described in gastric cancer (51). Furthermore, we noted that MAIT cells can co-express inhibitory receptors NKG2A and PD-1, at levels similar to CD8 T cells (Figure 3H), meaning that MAIT cells are potentially targetable by immune checkpoint inhibitor therapies (e.g., monalizumab and pembrolizumab, respectively), both alone and in combination. Pembrolizumab has recently been approved by the US FDA to treat patients with advanced cancers of the gastroesophageal junction (52), therefore, it is becoming increasingly important to understand the impact of such treatment on the function of different T cell subsets. No differences in PD-1 expression were detected in a preliminary analysis comparing *n* = 4 unmatched pre-treatment and post-treatment tumors (data not shown). This differs from earlier observations by our colleagues, who note a loss of PD-1 in OAC tumor T cells post-treatment (53), however greater numbers of patients are needed to confirm this finding, ideally in matched patient samples at pre- and post-treatment time points. MAIT cells have previously been reported to be exempt from the deleterious effects of chemotherapy, unlike other T cell subsets (3).

Functional analyses showed that MAIT cells freshly isolated from healthy donors produced less IFN-γ and TNF-α when activated in the presence of tumor conditioned media prepared from stage III OAC tumors (Figure 4). The observed decrease in production of these important anti-tumor cytokines by activated MAIT cells has also been reported in liver cancer (54), colorectal cancer (17), or after exposure to colorectal TCM (14). Colorectal studies have also reported a concomitant elevation in MAIT cell expression of IL-17 (15, 17). The role of pro-inflammatory cytokine IL-17 in tumors is controversial (55), but its abundance in colorectal tumors has been linked with negative prognostic outcomes (56) suggesting that overall IL-17 plays a negative role, promoting pro-tumor inflammation, angiogenesis, and metastasis (57). We did not observe any change in IL-17A expression in our experiments. We also observed that expanded MAIT cells were capable of killing an oesophageal cancer cell line, in agreement with similar reports in colorectal cancer (17) and multiple myeloma (18). We did not observe any impact of OAC TCM in this setting, though it is possible that the 4 h incubation time used was not sufficient to observe the effects of the TCM on cytoxicity. We used MAIT cells expanded from healthy blood donors to model the effects of the OAC tissue microenvironment. Our TCM experiments show that T helper (T_H_) – 1 cytokine production, but not cytotoxicity, is negatively affected *in vitro* by the soluble tumor microenvironment. Whether the observed effects of the OAC TCM are tumor-specific remains to be determined, as we were unable to obtain normal oesophageal tissue to control for tissue effects. Intriguingly, previous work by our group has shown that conditioned media from OAC and BO explants, but not normal or oesophagitis, was able to reduce IFN-γ and TNF-α production in CD8 T cells, suggesting that this immunosuppression may arise early in OAC development (58). We hypothesize that, as reported for other effector cells, MAIT cell function is at least partially subverted in the OAC tumor microenvironment toward a pro-tumor phenotype, which may potentially be amenable to rescue by immune checkpoint inhibitor therapy (59). Furthermore, MAIT cells are well-represented in the pre-neoplastic BO lesion and if they indeed are proven to have immunosuppressive phenotypes at this stage, it is also feasible that these cells could be targeted prior to cancer development, as a preventive measure. Indeed, MAIT cell presence in tumors has been associated with poor survival outcome in small cohort studies (15, 17). If MAIT cells truly are linked with negative outcomes, then these cells pose a potential confounding factor for prognostic studies focussed on CD8^+^ T cells, perhaps explaining why not all studies demonstrate a clear link between CD8^+^ TIL levels and survival (60). This calls for future, larger studies to differentiate between the prognostic ability of these cell types, to assess whether MAIT cells should be excluded to improve prognostic scoring approaches.

We observed that MAIT cell frequencies in OAC blood and tumors were negatively correlated with serum levels of the chemokine IP-10, a pro-inflammatory chemoattractant for activated T cells. This finding is in agreement with a study on cardiometabolic disease which reported a consistent inverse correlation between MAIT cells and IP-10 expression (61). MAIT levels in tumors were also inversely correlated with chemokines MDC and MIP-1β. MDC is expressed by tumor cells and tumor-associated macrophages, and is responsible for recruitment of regulatory T cells to the tumor site (62). MAIT cells in circulation have been shown to express receptors for IP-10 (CXCR3) (63) and CCL22 (CCR4) (64), respectively, rendering them receptive to these chemokines, however, what this means for anti-tumor immunity is currently unclear.

Oesophageal adenocarcinoma tumors have a relatively high mutational burden (65), are a rich source of tumor neo-antigens and are generally well-infiltrated by immune cells. Indeed, histological analysis of haemotoxylin and eosin stained biopsies from our OAC biopsy cohort revealed an overall high level of immune cell infiltration and low (<50%) percentage of tumor stroma in the majority of cases (manuscript in preparation). Such features would suggest that OAC tumors should respond well to immune checkpoint inhibitor treatment (66, 67), yet results to date have been modest, with only a minority of patients benefitting from treatment (68, 69). Interestingly, gene signatures correlating with clinical response to immune checkpoint inhibitor treatment highlight the importance of IFN-γ and its related genes, most notably CD8A, IP-10, and HLA-DR (68–70). We and others have demonstrated a prognostic role for the class II antigen presentation molecule HLA-DR in OAC (71–73). Therefore, although T cell presence in tumors is evidently advantageous (37), it must be accompanied by effective T cell activation machinery, and recent reviews have argued that effective T cell priming should be considered as much as immune checkpoint expression (74). This raises questions about the role of MR1, the MAIT cell antigen presentation molecule, in tumors. We could not detect MR1 expression on OAC tumor cells, either in fresh collagenase-digested biopsies, or from trypsinised OE33 cell lines (data not shown), whereas Gherardin and colleagues demonstrate MR1 upregulation in a K562 cell line after pulsing with folate-derived antigens (18).

Future studies are required to assess the relative contribution of MAIT cells to the overall anti-tumor immune response *in vivo*, and the impact of immune checkpoint inhibitor treatments on the function of different CTL types. Elucidation of MAIT cell tumor immunology is of particular interest for a number of reasons. Firstly, we and others highlight an anti-tumor cytotoxic capability for MAIT cells, at least *in vitro* (17, 18). If immune checkpoint inhibitors can abrogate the suppressive effects of the tumor microenvironment on MAIT cell function, these cells themselves may have important therapeutic potential, as has been proposed for other innate T cells (19, 75). Secondly, MAIT cells naturally express high levels of the multi-drug efflux protein ABCB1, which confers resistance to the deleterious effects of chemotherapy (3), meaning that MAIT cell contributions to anti-tumor immunity could be particularly prominent in the combination treatment setting, where chemo-sensitive CTLs are killed off. We noted no difference in MAIT cell frequency after neo-adjuvant chemotherapy or chemoradiotherapy treatment, when compared to an unmatched cohort of treatment-naive patients. Further analysis is warranted to confirm this observation however, ideally in the same OAC donors at pre- and post-treatment time points. And thirdly, recent studies show that the gut microbiome plays an important role in the optimal efficacy of immune checkpoint inhibitors (76). Since MAIT cells are sensitive to the microbial milieu, relying on gut flora for development (77), it stands to reason that gut microbes may also affect MAIT cell function in the cancer setting. These observations raise interesting questions regarding the dynamic immune response to cancer—and potentially offer insights into new ways in which it may be best targeted therapeutically. As immunotherapeutic treatment of gastrointestinal cancers becomes more common, there is a growing urgency to re-evaluate what is known about the immune response to cancer, and to learn more about the basic biological processes required for an optimal tumor response. A greater understanding of MAIT cell functions in cancer will aid this expanding knowledge base, and improve our understanding of innate T cells, a cell type with great unrealised immunotherapeutic potential.

## Data Availability

The datasets generated for this study are available on request to the corresponding author.

## Ethics Statement

Ethical approval was granted from the St. James's Hospital and Adelaide, Meath and National Children's Hospitals Research Ethics Committee (SJH/AMNCH, reference number 041113/10804). All specimens were collected with prior informed consent, from patients attending St. James's Hospital or from healthy age-matched donors. This study was carried out in accordance with the World Medical Association's Declaration of Helsinki guidelines on medical research involving human subjects.

## Author Contributions

MD conceived the study, designed all experiments, performed experiments and analyses, and drafted paper. AM performed cytotoxicity experiments and phenotyping of post-treatment specimens. AO performed cytokine experiments. JP and SK performed statistical analyses and data correlations. NW, NV, GB, and AH were responsible for generation of expanded MAIT cells. NC and EF were responsible for specimen and clinical data collection and management. AR was responsible for compiling clinical follow-up data. FM, DO, NR, and JR responsible for specimen acquisition. MC, AH, and JO advised the study, helped devise experiments, and critically evaluated manuscript drafts. All authors contributed to the drafting, critical analysis, and editing of this manuscript.

### Conflict of Interest Statement

The authors declare that the research was conducted in the absence of any commercial or financial relationships that could be construed as a potential conflict of interest.
